# Developing a deep learning model for the automated monitoring of acupuncture needle insertion: enhancing safety in traditional acupuncture practices

**DOI:** 10.1186/s12906-025-04853-7

**Published:** 2025-03-18

**Authors:** Shun-Ku Lin, Chien-Kun Su, Melnard Rome C. Mercado, Syu-Jyun Peng

**Affiliations:** 1 Department of Chinese medicine, Renai Branch, Taipei City Hospital, Taipei, Taiwan; 2 Institute of Traditional Medicine, School of Medicine, National Yang Ming Chiao Tung University, Taipei, Taiwan; 3 Institute of Public Health, School of Medicine, National Yang Ming Chiao Tung University, Taipei, Taiwan; 4 University of Taipei, Taipei, Taiwan; 5 Department of Electrical Engineering, Chung Hua University, Hsinchu, Taiwan; 6 In-Service Master Program in Artificial Intelligence in Medicine, College of Medicine, Taipei Medical University, No.250, Wuxing St., Xinyi Dist, Taipei City, 110 Taiwan; 7 Clinical Big Data Research Center, Taipei Medical University Hospital, Taipei Medical University, Taipei, Taiwan

**Keywords:** Acupuncture needle, Needle detection, Deep learning, Safety, Acupuncture practice

## Abstract

**Background:**

Acupuncture is a widely practiced traditional therapy, yet safety concerns, particularly needle breakage and retention, remain critical issues that can lead to complications such as infections, organ injury, or chronic pain. This study aimed to develop a deep learning model to monitor acupuncture needle insertion, detect instances of needle breakage, and prevent needle retention, ultimately improving patient safety and treatment outcomes.

**Methods:**

A deep learning model based on the YOLOv8 architecture was trained using a dataset comprising 192 images from a commercial image library and 73 clinical images captured during real-world acupuncture sessions. Images were preprocessed through cropping and annotation, and augmented to enhance model generalizability. Five-fold cross-validation was employed to ensure robust performance. Model evaluation metrics included precision, recall, F1 score, and mean average precision (mAP) at Intersection over Union (IoU) thresholds of 50% (mAP@50) and 50–95% (mAP@50–95).

**Results:**

The model demonstrated strong performance, achieving an average precision of 88.0% and a recall of 82.9%. The mean average precision was 88.6% at mAP@50 and 62.9% at mAP@50–95, indicating high reliability in detecting acupuncture needles across diverse scenarios. These results highlight the potential of the model to enhance clinical safety by minimizing risks associated with needle breakage and retention, regardless of practitioner experience or patient demographics.

**Conclusions:**

The proposed YOLOv8-based deep learning model offers a reliable method for real-time needle monitoring in acupuncture. Its integration into clinical workflows can improve safety and efficiency, especially in underserved regions or settings with less experienced practitioners. Future research should validate the model with larger, more diverse datasets and explore its application in various healthcare settings.

**Trial registration:**

Not applicable; this study did not involve a healthcare intervention requiring registration. Data collection adhered to ethical standards with institutional approval (TCHIRB-11310004).

**Supplementary Information:**

The online version contains supplementary material available at 10.1186/s12906-025-04853-7.

## Introduction

Acupuncture is widely used in managing pain and enhancing overall well-being [1]; however, safety concerns persist [2]. There is particularly concern regarding the breakage of needles and retention of needle fragments in the body, which can lead to infection, organ injury, or chronic pain [3, 4].

The recent adoption of artificial intelligence (AI) methods to automate needle tracking has proven highly effective in acupoint selection and outcome prediction. It has also been shown to enhance safety by increasing the likelihood of detecting needle fragments under the skin surface [5]. AI can help to ensure consistency in technique transfer and reduce reliance on practitioner experience [6, 7]. AI technology also addresses the need for standardized evidence-based practices to enhance the effectiveness of procedures, while minimizing the associated risks [8].

Object detection models, such as those based on the YOLO framework, have proven effective in detecting tumors and abnormalities in radiological images [9]. However, researchers have yet to develop a model specifically for the detection of acupuncture needles fragments, while allowing seamless integration within the acupuncture workflow to facilitate real-time diagnostics.

Researchers have recently employed deep learning models, such as the Oriented R-CNN, to identify and count elongated objects (e.g., acupuncture needles). The oriented box detection scheme helps to enhance precision and accuracy, while the cloud-based operations ensures high computational efficiency [5]. This approach has proven effective in the counting of needles in standardized environments, such as the placement of needles in blue kidney dishes; however, it is ill-suited to the real-time detection of needles under the skin surface during clinical procedures.

Researchers have identified significant disparities in the quality of acupuncture care and access to skilled practitioners across different sociodemographic groups. It is plausible that patients in underserved regions face an elevated risk of adverse events due to less stringent safety protocols or insufficiently trained practitioners. Our objective in this study was to ensure a high standard of care for all patients, regardless of the physician’s level of experience [5]. This was achieved by developing a deep learning model based on YOLOv8 to automate the real-time detection of broken or misplaced acupuncture needles during treatment.

## Methods

### Study design

This study followed a cross-sectional design aimed at capturing real-world scenarios through the collection of clinical images during acupuncture sessions. The primary objective was to ensure that the needles were correctly placed and safely removed post-treatment. The research protocol was approved by the Taipei City Hospital Research Ethics Committee under approval number TCHIRB-11,310,004.

### Data

The dataset used in this study included images from the publicly available Roboflow dataset, which consists of 192 images showing acupuncture needles in bundles as well as after placement in patients. Note that this dataset covers a diverse range of needle insertion scenarios to enable robust detection. We also employed a dataset of 73 images captured during real-time acupuncture treatments in a clinical setting. The rationale for using two datasets was to ensure that the model is representative of real-world acupuncture practices, covering a wide range of needle sizes, insertion techniques, and patient demographics (e.g., skin tones).

The clinical data were anonymized to protect patient privacy, and no follow-up was required since the study focused solely on the detection of needles during the treatment session.

The sample size for this study was based on model training requirements, dataset diversity, and insights gained from previous research on object detection tasks. Considering the challenges in detecting acupuncture needles in real-world settings, our aim was to compile a sufficiently large dataset to ensure robust model performance while maintaining practical feasibility. The dataset included 192 images from the publicly available Roboflow dataset and 73 clinical images captured during real-world acupuncture procedures. The inclusion of clinical images was intended to enhance the generalizability of the model across diverse needle insertion conditions. The final sample size was meant to balance computational efficiency with detection accuracy, in accordance with the insights provided by previous studies employing deep learning models for medical image analysis. Five-fold cross-validation was conducted to optimize model performance and reduce the risk of overfitting.

### Participants

This study was conducted in a clinical acupuncture setting representative of both primary and secondary care environments. Study participants included 73 individuals undergoing standard acupuncture treatments at a designated acupuncture facility (anonymized for privacy reasons). Images were captured in real time during acupuncture sessions. All participants were adults (18 years and older), with no restrictions based on gender or ethnicity. Patients with known allergies to acupuncture needles or with conditions contraindicating acupuncture were excluded.

Note that no treatments were administered specifically for the purposes of this study. Data collection was centered around standard acupuncture procedures conducted during routine clinical practice. Efforts were made to ensure consistency in treatment regimens during both the development of the deep learning model and its subsequent evaluation. The primary objective of this study was to detect acupuncture needles during routine treatments without interfering with the therapeutic process.

### Data preparation

Data preprocessing was performed to enhance the quality of the images and thereby improve the accuracy of the deep learning model. This involved cropping images to remove background noise in order to focus on the area of interest where the needles were inserted. The cropped size was standardized to 640 × 640 pixels to ensure consistency across the dataset.

The Imaug library was used to perform image augmentation to mitigate the effects of a small training dataset, thereby preventing overfitting and improving the generalizability of the model. Augmentation involved horizontal and vertical flipping, random rotations within a range of -40° to 40°, and brightness adjustments from 50 to 130%. The transformations resulted in four variations of each original image.

### Manual annotation

Manual annotation was performed by two domain experts with extensive experience in acupuncture and medical imaging analysis. The results were independently reviewed by a senior physician to ensure accuracy. The annotation process involved bounding box labeling of acupuncture needles in each image using the software tool, Labeling. The annotation criteria were pre-defined according to needle visibility, orientation, and insertion depth to ensure consistency.

Annotation reliability was assessed by conducting inter-annotator agreement analysis using Cohen’s kappa coefficient, which indicated strong agreement between annotators. To resolve instances of disagreement, a third expert reviewed the annotations and made the final decision. To ensure quality control, we implemented a multi-step verification process involving multiple review rounds to identify and correct potential errors, such as mislabeling, inconsistent bounding boxes, or partial occlusions. Considering the expertise required for accurate needle identification, we opted against crowdsourcing or automated annotation methods.

### Outcomes

The primary outcome predicted by the deep learning model is the accurate detection of acupuncture needles following insertion. The design of the proposed system is based on the need to ensure that needles are properly placed and not left in the patient after the session. The time horizon for this prediction covers the duration of the acupuncture session, from needle insertion to removal. Outcomes are continuously assessed throughout the treatment process, enabling immediate feedback to the acupuncturist in the event that any needles are unaccounted for after the procedure.

The rationale for selecting needle detection as the primary outcome was to ensure patient safety. Retained needles can result in complications such as infection, organ damage, or chronic pain. Outcome assessments were performed using the same methods and the same standardized images for all sociodemographic groups, regardless of the patient’s skin tone or other physical characteristics. Automating the outcome assessment eliminated the subjective interpretation typically required of human assessors, thereby reducing the qualifications needed for outcome evaluation.

### Predictors

The selection of predictors for the model was based on a review of the literature and the specific requirements for detecting acupuncture needles in a clinical setting. The primary predictors included image features, such as the size and shape of the acupuncture needles, insertion angle, and color contrast. These predictors were identified as the most important visual characteristics for achieving detection accuracy. Many object detection models developed for medical imaging rely on similar visual features.

To ensure accuracy, the predictors were extracted from annotated images that had undergone manual labeling. Image features were captured from the time of needle insertion and throughout the procedure. Consistency in timing across all images allowed the model to detect the needles regardless of the treatment stage (i.e., during insertion as well as after insertion). To minimize potential biases, the same methods for cropping, augmentation, and predictor assessment were consistently applied to all images, regardless of the sociodemographic group.

### Analytic methods

The collected data were used to both develop and test the deep learning model for acupuncture needle detection. This involved partitioning the dataset into training and test sets using K-fold cross-validation, ensuring that the entire dataset contributed to both training and testing at different stages. This approach maximized the use of a limited dataset and minimized the risk of overfitting. Data augmentation was also applied to improve the generalizability of the model by introducing a wider range of training image variables, such as rotation, flipping, and brightness adjustments.

The primary predictors were the visual features of the acupuncture needles, including their shape, size, and orientation. These predictors were rescaled to a standardized size to enhance learning efficiency, and the deep learning model automatically extracted the features, eliminating the need for manual interpretation.

We selected the YOLOv8 model for this study due to its ability to detect objects with a high degree of precision in real-time. Hyperparameter tuning involved adjusting the learning rate, batch size, and augmentation parameters. An internal test of the model was performed using K-fold cross-validation to ensure that performance metrics were robust across various data subsets. Model performance was assessed using several key metrics, including precision, recall, F1 score, and mean average precision (mAP) at different Intersection over Union (IoU) thresholds (mAP@50 and mAP@50–95).

### Evaluating model performance

The outputs of the proposed prediction model include an indication of the presence (or absence) of an acupuncture needle in the image, as well as its specific location indicated by a bounding box. The model also provides a confidence score indicating the probability that the detected object is indeed an acupuncture needle. The final classification threshold was determined by analyzing performance metrics across a range of thresholds during model training and testing.

We selected an Intersection over Union (IoU) threshold of 0.5, which represents the point where the overlap between predicted and actual bounding boxes is 50%. The mAP@50 metric calculated at this threshold demonstrates the effectiveness of the model in detecting acupuncture needles across various images. The mAP@50–95 metric evaluates performance across multiple IoU thresholds (from 50 to 95%), providing a more comprehensive measure of model accuracy across different levels of detection difficulty.

The rationale for selecting these thresholds was to balance precision (correctly identified needles) and recall (sensitivity in detecting all possible needles), thereby ensuring robust and reliable needle detection across a range of clinical settings.

## Results

### Participants

The 73 participants in the clinical dataset underwent standard acupuncture procedures involving the placement of needles in specific acupoints during which images were captured for analysis. No follow-up was performed as the study focused on needle detection during the procedure. There were no issues pertaining to missing data as the images were carefully curated and preprocessed prior to inclusion. A comparison of the development and evaluation datasets revealed consistency in the predictors; however, the clinical dataset encompassed a wider range of real-world conditions compared to the Roboflow dataset.

### Performance evaluation: internal test set

Model development was performed using 192 images from the Roboflow dataset and 73 from the clinical dataset. The YOLOv8 model was trained and tested using 5-fold cross-validation to ensure robust performance across various data subsets. In each fold, the performance of the model in detecting acupuncture needles was assessed in terms of precision, recall, F1 score, and mean Average Precision at mAP@50 and mAP@50–95. The detailed performance metrics of the fine-tuned YOLOv8 model on the internal test set are summarized in Table 1.

During the 1st fold of training, the model achieved precision of 81.50%, recall of 67.20%, and an F1 score of 73.66%. The mean average precision at an IoU threshold of 50% (mAP@50) reached 73.70%, while the mAP across a range of IoU thresholds from 50 to 95% (mAP50-95) was 45.30%. These metrics highlight the model’s performance after 99 epochs of training, showing steady improvements across precision, recall, and mAP values as training progressed. Supplementary Fig. 1 illustrates these performance metrics over time, with precision, recall, mAP@50, and mAP50-95 improving significantly through the training process.

In the 2nd fold of training, the model performance improved, with precision of 85.90% and recall of 75.70%, yielding an F1 score of 80.47%. The mAP@50 increased to 85.20%, and the mAP50-95 reached 63.20%, indicating more robust performance across different IoU thresholds compared to the 1st fold. These metrics suggest a better balance between precision and recall during this fold, which is further visualized in Supplementary Fig. 2, where all performance metrics display consistently upward trends through each epoch.

In the 3rd fold, the model achieved precision of 90.10% and recall of 89.90%, resulting in an F1 score of 89.99%. The mAP@50 metric reached 92.80%, while the mAP50-95 increased to 65.50%, demonstrating excellent model performance. Supplementary Fig. 3 shows the steady increase in these metrics throughout the training process, reflecting the model’s high accuracy in detecting acupuncture needles with consistent overlap between predictions and actual objects.

During the 4th fold, the model continued to perform well with precision of 88.60%, recall of 88.30%, and an F1 score of 88.44%. The mAP@50 metric reached 94.00% and the mAP50-95 increased to 67.20%. These results indicate that the model maintained a strong balance between precision and recall while improving its ability to detect objects at varying IoU thresholds. As shown in Supplementary Fig. 4, these metrics showed steady improvements through the epochs.

In the 5th and final fold, the model achieved its best performance with precision of 93.90% and recall of 93.20%, leading to an F1 score of 93.54%. The mAP@50 reached 97.20%, while the mAP50-95 increased to 73.30%, highlighting the strong generalizability and accuracy of the model. Supplementary Fig. 5 captures these performance metrics, demonstrating and the capability of the model to accurately detect acupuncture needles under diverse conditions following the culmination of the training process. During the model training process, performance metrics were evaluated across five folds, each of which spanned 99 epochs.

Supplementary Fig. 1 depicts the first fold, where precision, recall, mAP@50, and mAP50-95 steadily improved over time. The x-axis indicates the training epoch, while the y-axis displays the metric values ranging from 0 to 1. The blue, orange, cyan, and pink lines respectively indicated mAP@50, mAP50-95, precision, and recall, illustrating a consistent upward trend. Supplementary Fig. 2 depicts the second fold, where significant gains in precision and recall were observed. Supplementary Fig. 3 depicts the third fold, showing sustained increases in mAP values and consistently high precision and recall. Supplementary Fig. 4 depicts the fourth fold, highlighting a strong balance between precision and recall, with further improvements in mAP@50 and mAP50-95 as training progressed. Supplementary Fig. 5 depicts the fifth fold with the highest peak precision, recall, and mAP values.

When applied to the internal dataset, the average performance of the model was robust across all five folds with mean precision of 88.0% (SD 4.6%), mean recall of 82.9% (SD 11.0%), mean F1 score of 85.2% (SD 8.0%), mean mAP@50 of 88.6% (SD 9.4%), and mean mAP@50–95 of 62.9% (SD 10.5%).

**Table 1:** Performance evaluation of fine-tuned YOLOv8 model applied to internal test set using 5-fold cross-validation

Fold	Precision	Recall	F1 Score	mAP@50	mAP@50–95
1	81.5%	67.2%	73.7%	73.7%	45.3%
2	85.9%	75.7%	80.5%	85.2%	63.2%
3	90.1%	89.9%	90.0%	92.8%	65.5%
4	88.6%	88.3%	88.4%	94.0%	67.2%
5	93.9%	93.2%	93.5%	97.2%	73.3%
Mean (SD)	88.0 (4.6)%	82.9 (11.0)%	85.2 (8.0)%	88.6 (9.4)%	62.9 (10.5)%

### Performance evaluation: external test set

Model performance was also evaluated using an external test dataset, with precision, recall, and mAP measured at an IoU threshold of 0.50 and a confidence threshold of 0.45.

The average accuracy in this analysis was 79.16%, with six instances of misclassification and two missed detections. Table 2 summarizes the performance on individual test images. The lowest average accuracy was observed for Image 01 (66.40%, with one missed detection), which may be attributed to occlusion or lighting issues. The highest average accuracy was obtained for Image 09 (92.78%).

**Table 2:** Classification accuracy of YOLOv8 model applied to external test set of 10 images. The right column indicates misclassified images and those with missed detections indicating areas requiring further model improvement

Images	Accuracy	Note
01	66.40%	1 missing
02	84.69%	
03	85.72%	
04	78.42%	
05	77.01%	
06	78.42%	2 misclassified
07	77.14%	3 misclassified
08	78.78%	1 misclassified & 1 missing
09	92.78%	
10	72.20%	

Total Images: 10; Mean (SD): 79.16 (7.3)% 6 misclassified and 2 missing

**Fig. 1:**
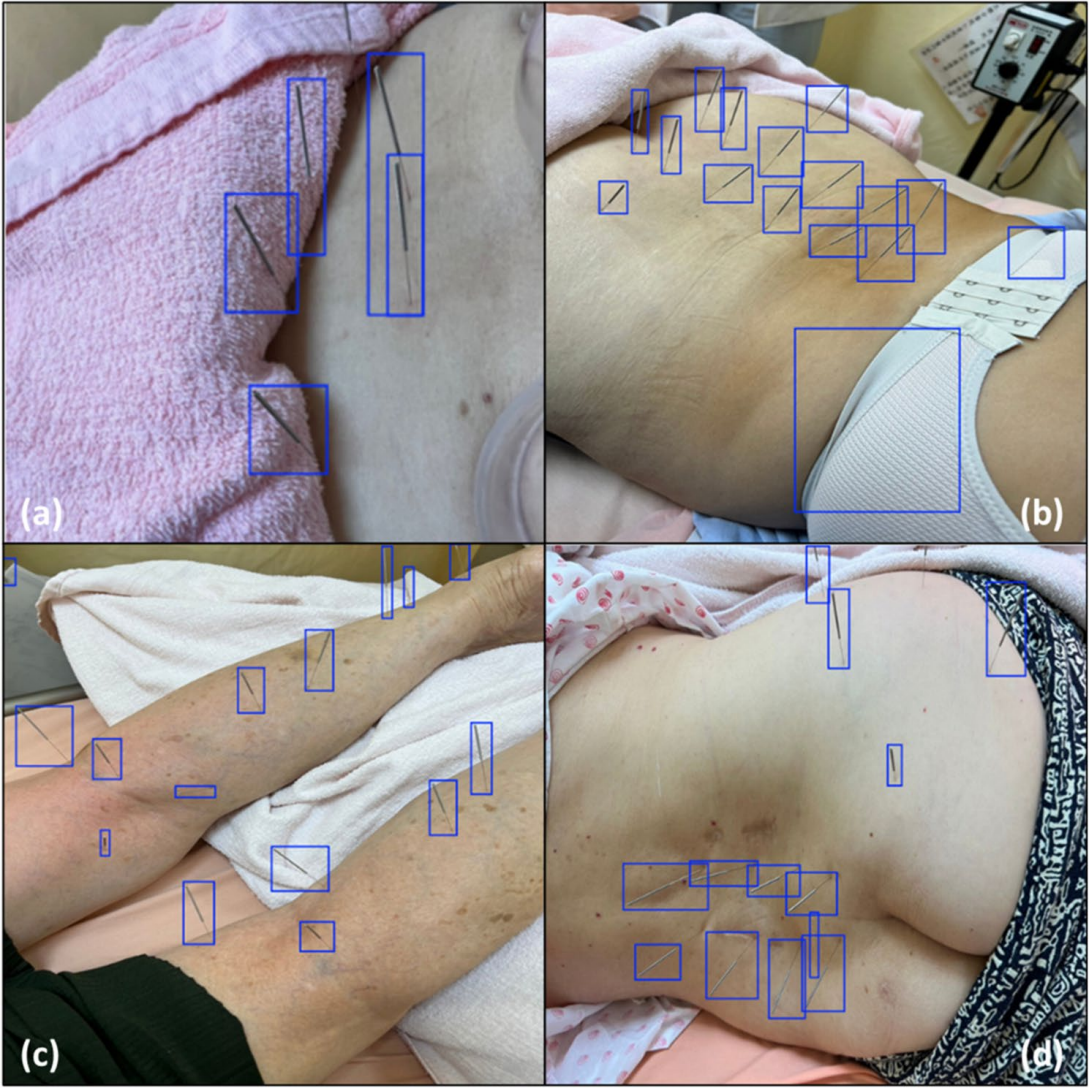
Instances of misclassification and missing detection when assessing the performance of the proposed model using an external test dataset. This visual comparison of the ground truth and predicted bounding boxes illustrates the challenges typically encountered in needle detection, such as occlusion or a lack of contrast between the needles and the background. The figure includes the following: (**a**) Image 01 with 1 missed detection; (**b**) Image 06 with 2 misclassified detections; (**c**) Image 07 with 3 misclassified detections; and (**d**) Image 08 with 1 misclassified detection and 1 missed detection

Despite the overall good performance of the model, instances of misclassifications and missed detections were identified. The misclassification of needles in Images 06 and 07 highlights areas for fine-tuning. Figure 1 presents instances of misclassification and missed detections, offering insight into areas for model improvement.

## Discussion

### Interpretation

The overall results indicate that our YOLOv8-based deep learning model was highly effective in detecting acupuncture needles beneath the skin under various conditions, as evidenced by precision of 88.0% and recall of 82.9% across multiple internal test folds. mAP scores of 88.6% (at IoU 50%) and 62.9% (at IoU 50–95%) indicate the consistency of the model across detection thresholds. These results indicate that the proposed model could enhance the safety of patients in any clinical settings, regardless of practitioner experience or regional healthcare resources. This is particularly relevant in underserved areas where safety protocols may not be as rigorous.

The existing Oriented R-CNN model is meant to reduce human error in the manual counting of needles through the use of oriented object detection, which is particularly effective when dealing with elongated objects (e.g., acupuncture needles) in a controlled setting [5]. That model demonstrated high accuracy, precision, and recall when applied to the counting of needles as well as high efficiency when integrated with a cloud-based system and Telegram bot.

The Oriented R-CNN and YOLOv8 models are both meant to keep a tally of acupuncture needles; however, there are notable differences in their objectives and the complexities involved. The Oriented R-CNN is meant to count needles in a standardized vessel (a blue kidney dish), whereas our YOLOv8 model is meant to detect needles inserted into the skin at various angles, against backgrounds of various skin tones in images captured at various distances.

Growing interest in acupuncture highlights the potential for adverse events (AEs), ranging from bruising, mild pain, or dizziness to cardiac tamponade, nerve injury, or infection [10, 11]. In a systematic review, Bäumler et al. (2021) reported that 9.31% of patients experienced at least one AE, most of which were minor. Nonetheless, rare but serious AEs highlight the need for vigilance [12].

Several studies conducted in Taiwan provide further insight into specific risks. Lin et al. (2019) reported a relatively high incidence of cellulitis (64.4 per 100,000 procedures), particularly among patients with diabetes, chronic kidney disease, or varicose veins [13]. Researchers have also reported a relatively high incidence of acupuncture-related nerve injury (5.76 per 10,000 procedures) correlated to age and the presence of chronic conditions [14]. These findings underline the importance of individualized patient assessments, particularly when treating patients with pre-existing health conditions.

The risk of AEs also indicates the need for standardized protocols, including improved reporting mechanisms and safety guidelines. It has been suggested that the true risk profile of acupuncture may be skewed due to the underreporting of mild AEs, such as needle retention [10]. Park et al. (2016) emphasized the need for specialized training to deal with complications such as hypersensitivity reactions and infectious skin diseases [11].

The transformation of traditional Chinese medicine (TCM) by AI technology has had a profound impact on clinical precision and therapeutic efficacy. AI-driven systems have been developed to address various aspects of acupuncture, including diagnosis, treatment prescription, manipulation tracking, and the prediction of therapeutic outcomes [6]. AI-based data mining has also made it possible for practitioners to identify effective acupoint combinations based on rigorous scientific analysis, effectively replacing experience with evidence-driven protocols [7]. This shift has also made it possible for clinicians to predict patient responses to facilitate screening [8]. Finally, the application of AI to computer vision provides an objective evaluation of acupuncture manipulations to ensure the precise transfer of techniques between practitioners and foster skill inheritance.

AI-guided acupuncture is poised to revolutionize traditional practices by advancing evidence-based practice, predictive medicine, and personalized health management [7]. Nonetheless, there is a pressing need for further model interpretation, a robust data infrastructure, and the harmonization of ancient philosophical concepts with modern AI frameworks [6].

### Usability of the proposed model in the context of current care

From the current care perspective, the usability of the YOLOv8 model for acupuncture needle detection depends largely on the quality and availability of input data. Poor-quality images with low contrast, improper lighting, or obstructions can significantly impair detection accuracy. Image preprocessing to adjust brightness, contrast, and resolution is essential. The system should also include protocols to discard unusable images or flag them for manual review by healthcare professionals.

System automation can relieve healthcare professionals from the need to perform data preprocessing; however, users are still required to capture high-quality images under clinical conditions. This generally necessitates basic training in lighting, proper framing, and focusing techniques.

Future research should explore the applicability and generalizability of the model across diverse clinical environments. Training datasets should be expanded to include a wider diversity of patients, needle types, and insertion techniques. Researchers could also investigate the model’s applicability when integrated with real-time clinical workflows and assess its utility in various healthcare settings.

### Clinical value and practicality

The findings of this study underscore the potential clinical value of AI methods in the detection of acupuncture needles. By automating the monitoring process, the proposed YOLOv8-based model could alleviate the repetitive and labor-intensive tasks associated with manual needle verification. This would be particularly beneficial in high-volume acupuncture clinics and hospital settings, where practitioners must consistently ensure the accurate placement and removal of needles. Automating this process could reduce the cognitive load of healthcare providers, allowing them to devote greater attention to patient care rather than performing constant visual inspections.

The proposed technology enhances workflow as well as the safety of patients and practitioners. Real-time AI-assisted alerts can significantly reduce the risk of needle retention, breakage, or loss, thereby preventing complications such as infections, organ injuries, and neuropathic pain. The proposed system provides consistent monitoring based on objective indicators, ensuring a standardized level of safety across practitioners, regardless of their skill levels. This is particularly valuable in training environments, where novice acupuncturists benefit from additional guidance and oversight.

From a broader healthcare perspective, this deep learning model aligns with modern clinical demands for efficiency and precision. By reducing reliance on human-dependent verification and minimizing preventable errors, the system can improve patient outcomes while optimizing resource utilization. Future research should prioritize real-world implementation strategies, such as integrating the model with handheld imaging devices or automated clinical documentation systems, to further improve its practicality in clinical acupuncture settings.

### Annotation bias and future directions

Manual annotation carries an inherent risk of bias, particularly when defining ambiguous needle positions, assessing variations in insertion depth, or distinguishing overlapping needles. Expert annotation can help to ensure high accuracy and strong inter-annotator agreement; however, subtle inconsistencies in boundary definitions undermine generalizability. Note that systematic bias in annotation—such as a tendency to label clearly visible needle boundaries while underrepresenting occluded or low-contrast needles—could reduce the sensitivity of the model in clinical practice.

To mitigate these limitations, future research should explore semi-automated annotation techniques that incorporate edge-detection algorithms and pre-trained object detection models to assist with bounding box placement. These methods could enhance labeling consistency by reducing subjectivity. It should also be possible to employ active learning strategies for the iterative refinement of the annotation process, prioritize the most challenging samples, optimizing both data quality and annotation efficiency, enabling the model to flag uncertain predictions and prompt expert reviews only for ambiguous cases.

By integrating these advanced annotation methodologies, future studies can further improve dataset reliability, reduce annotation workload, and enhance the robustness of deep learning models for acupuncture needle detection in clinical applications.

Future research should explore the application of recently developed models such as YOLO-V10 and YOLO-World. YOLO-V10 offers an advanced backbone with improved feature extraction capacity, which could further enhance detection precision. Meanwhile, YOLO-World supports zero-shot detection, potentially aiding detection in cases with limited datasets. Evaluating these models in the acupuncture domain could provide valuable insights and further improve the robustness of AI-assisted safety systems.

Our sample-level analysis revealed that model failures were often associated with low contrast between needles and background, occlusion, and suboptimal image quality. These issues suggest potential biases in our training data, such as an underrepresentation of diverse skin tones and complex clinical environments. Future work should focus on expanding the dataset to cover a broader range of patient demographics and developing domain adaptation techniques to enhance the model’s robustness across varied clinical settings.

### Limitations

This study has several limitations that should be considered when interpreting our findings. First, we dealt with a relatively small sample size (265 images), which limited the statistical power of the model and may have contributed to data overfitting. Despite our use of data augmentation techniques, our reliance on a small and potentially non-representative sample may have restricted the model’s ability to generalize across diverse patient populations and clinical scenarios.

Many public datasets, including the one used in this study, do not fully capture the variability seen in real-world clinical environments, particularly regarding skin tone, needle size, and insertion techniques. Moreover, results obtained under controlled conditions can lead to statistical uncertainty when applied in clinical environments with greater variability. In the future, researchers should compile larger datasets that more accurately represent the complexities encountered in real-world practice.

Another limitation was the lack of a mechanism to handle missing data. Although we did not encounter significant issues with missing images or annotations in this study, this situation does not reflect the incomplete data or poor image quality commonly found in real-world clinical settings. The relatively clean datasets used in this study may affect the model’s robustness and its ability to handle missing or suboptimal data inputs during actual clinical use.

## Conclusions

This paper presents a YOLOv8-based deep learning model to monitor acupuncture needle insertion, detect instances of needle breakage, and prevent the retention of needle fragments.

Our preliminary results are promising; however, further testing with a larger, more diverse dataset will be required to enhance generalizability across clinical settings.

## Electronic supplementary material

Below is the link to the electronic supplementary material.

**Supplementary Material 1**: **Supplementary Fig. 1**: Performance metrics during the 1st fold of model training, including precision, recall, mAP@50, and mAP50-95 over 99 epochs. The x-axis indicates the number of training epochs, while the y-axis indicates the corresponding values of each performance metric (ranging from 0 to 1). The blue line represents mAP@50, the orange line indicates mAP50-95, the cyan line shows precision, and the pink line tracks recall. The plot demonstrates steady improvements across all metrics throughout the training period.

**Supplementary Material 2**: **Supplementary Fig. 2**: Performance metrics during the 2nd fold of model training, including precision, recall, mAP@50, and mAP50-95 over 99 epochs. The x-axis indicates training epochs, and the y-axis denotes the values of each performance metric. The plot presents a clear upward trend, reflecting notable gains in precision and recall throughout the training process.

**Supplementary Material 3**: **Supplementary Fig. 3**: Performance metrics during the 3rd fold of model training, including tracking precision, recall, mAP@50, and mAP50-95 over 99 epochs. The x-axis indicates training epochs, while the y-axis indicates the performance values. The plot highlights consistently high precision and recall and a progressive increase in mAP values, indicating strong overall model performance in detecting acupuncture needles.

**Supplementary Material 4**: **Supplementary Fig. 4**: Performance metrics during the 4th fold of model training, including the progression of precision, recall, mAP@50, and mAP50-95 over 99 epochs. The x-axis indicates the number of training epochs, and the y-axis indicates the values of each metric. The plot demonstrates a good balance between precision and recall, with continual improvements in mAP@50 and mAP50-95 as training advanced.

**Supplementary Material 5**: **Supplementary Fig. 5**: Performance metrics during the 5th fold of model training, including precision, recall, mAP@50, and mAP50-95 over 99 epochs. The x-axis indicates training epochs, and the y-axis displays the values of each metric. This fold presents the the highest performance, with exceptionally high precision, recall, and mAP values.

## Data Availability

The datasets used and/or analyzed during the current study available from the corresponding author on reasonable request.
