# Supplementary figures and images for: Developing a deep learning model for the automated monitoring of acupuncture needle insertion: enhancing safety in traditional acupuncture practices
Source: BMC Complement Med Ther. 2025 Mar 18;25:108. doi: 10.1186/s12906-025-04853-7 (PMC11917098; doi:10.1186/s12906-025-04853-7)

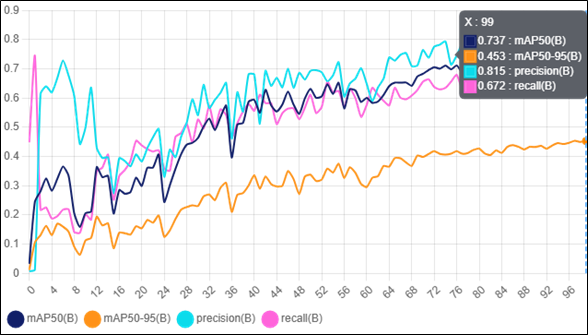

Supplement: Supplementary file 1 — Supplementary Material 1: Supplementary Fig. 1: Performance metrics during the 1st fold of model training, including precision, recall, mAP@50, and mAP50-95 over 99 epochs. The x-axis indicates the number of training epochs, while the y-axis indicates the corresponding values of each performance metric (ranging from 0 to 1). The blue line represents mAP@50, the orange line indicates mAP50-95, the cyan line shows precision, and the pink line tracks recall. The plot demonstrates steady improvements across all metrics throughout the training period. [file 12906_2025_4853_MOESM1_ESM.tif]

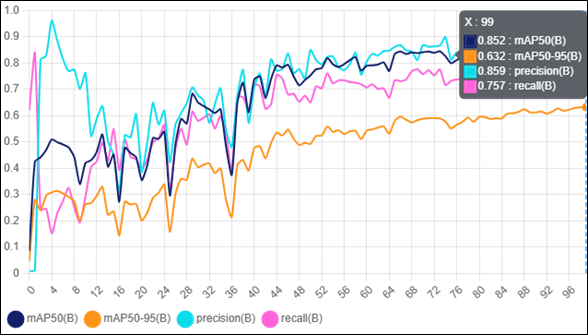

Supplement: Supplementary file 2 — Supplementary Material 2: Supplementary Fig. 2: Performance metrics during the 2nd fold of model training, including precision, recall, mAP@50, and mAP50-95 over 99 epochs. The x-axis indicates training epochs, and the y-axis denotes the values of each performance metric. The plot presents a clear upward trend, reflecting notable gains in precision and recall throughout the training process. [file 12906_2025_4853_MOESM2_ESM.tif]

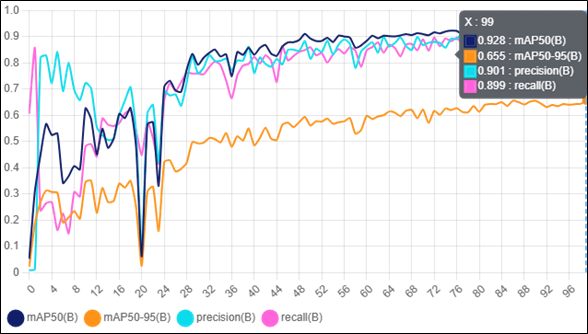

Supplement: Supplementary file 3 — Supplementary Material 3: Supplementary Fig. 3: Performance metrics during the 3rd fold of model training, including tracking precision, recall, mAP@50, and mAP50-95 over 99 epochs. The x-axis indicates training epochs, while the y-axis indicates the performance values. The plot highlights consistently high precision and recall and a progressive increase in mAP values, indicating strong overall model performance in detecting acupuncture needles. [file 12906_2025_4853_MOESM3_ESM.tif]

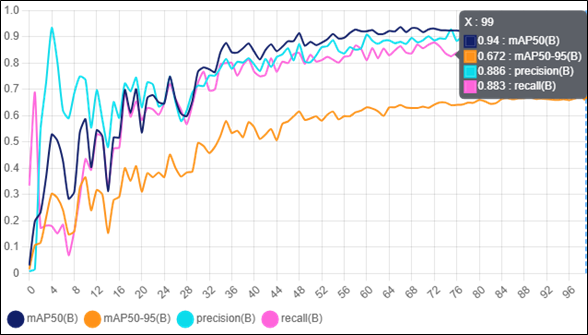

Supplement: Supplementary file 4 — Supplementary Material 4: Supplementary Fig. 4: Performance metrics during the 4th fold of model training, including the progression of precision, recall, mAP@50, and mAP50-95 over 99 epochs. The x-axis indicates the number of training epochs, and the y-axis indicates the values of each metric. The plot demonstrates a good balance between precision and recall, with continual improvements in mAP@50 and mAP50-95 as training advanced. [file 12906_2025_4853_MOESM4_ESM.tif]

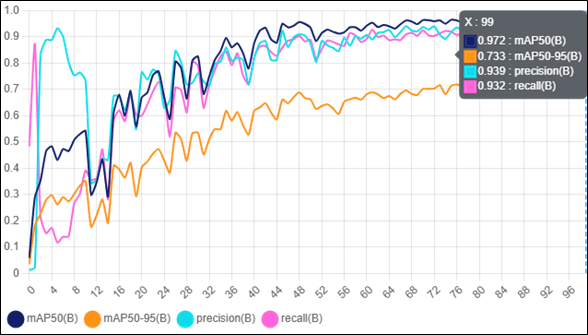

Supplement: Supplementary file 5 — Supplementary Material 5: Supplementary Fig. 5: Performance metrics during the 5th fold of model training, including precision, recall, mAP@50, and mAP50-95 over 99 epochs. The x-axis indicates training epochs, and the y-axis displays the values of each metric. This fold presents the the highest performance, with exceptionally high precision, recall, and mAP values. [file 12906_2025_4853_MOESM5_ESM.tif]
